# Excess body weight and its associated factors among first-year health sciences university students in Indonesia

**DOI:** 10.1371/journal.pone.0322773

**Published:** 2025-05-29

**Authors:** Dhanasari Vidiawati, Retno Asti Werdhani, Junaiti Sahar, Fitriana Murriya Ekawati, Linda Dewanti, Pudji Lestari, Endang R. Surjaningrum, Sulistiawati Sulistiawati, Indah Suci Widyahening

**Affiliations:** 1 Department of Community Medicine, Faculty of Medicine Universitas Indonesia, Jakarta,; 2 Primary Health Care Research and Innovation Center, Indonesia Medical Education and Research Institute, Faculty of Medicine Universitas Indonesia, Jakarta,; 3 Department of Community Health Nursing, Faculty of Nursing Universitas Indonesia, Depok,; 4 Department of Family and Community Medicine, Faculty of Medicine, Public Health and Nursing, Universitas Gadjah Mada, Yogyakarta,; 5 Department of Public Health Sciences-Preventive Medicine, Faculty of Medicine Universitas Airlangga, Surabaya,; 6 Faculty of Psychology Universitas Airlangga, Surabaya; United Arab Emirates University, UNITED ARAB EMIRATES

## Abstract

The incidence of overweight/obesity has been increasing among adolescents and young adults worldwide. University graduates majoring in health sciences are expected to become role models and agents of change in the future. Hence, the aim of this study was to understand the prevalence of excess body weight and risk factors among the university students in health sciences from the three biggest universities in Indonesia when they first entered the university. This cross-sectional study recruited all first-year students using a self-administered questionnaire and physical and laboratory examination data in 2022. The outcome was the prevalence of excess body weight, and the associated factors were students’ demographic characteristics, eating habits, and physical activities. Associated factors were measured by computing the odds ratio using logistic regression analysis. A total of 1,552 first-year students were included in the final analysis, and 77.6% were women. The prevalence of excess body weight was 34.7%. Independent factors, which increased the risk of excess body weight, were being male (adjusted odds ratio [AOR], 2.58; 95% CI 1.99–3.35), having a family history of obesity (AOR, 2.91; 95% CI 2.09–4.06), and eating <3 meals/day (AOR, 1.40; 95% CI 1.10–1.80). The prevalence of excess body weight among first-year health sciences university students in Indonesia was high. The health sciences faculties should design a curriculum which not only ensures that the students can provide health care to the communities in the future but also apply the knowledge to improve their health.

## Introduction

Overweight/obesity is a worldwide problem, and the number of adolescents and young adults who are overweight or obese is increasing globally [1]. Overweight and obesity are among the key risk factors for many non-communicable diseases (NCDs), such as coronary heart disease, stroke, and type 2 diabetes [2]. With the increasing burden of NCDs, which are the global leading cause of death, educational entities, such as universities, could play a more important role in its control through health promotion.

Studies among university students in several Asian countries reported an alarmingly high prevalence of overweight and obesity, which in some countries, including India, Pakistan, Kuwait, and Turkey, reaches more than 30% [3–12]. Studies on the health of health sciences university students, a specific demographic expected to bear more responsibility to improve a nation’s health, are likewise restricted. According to research from Peru and Saudi Arabia, the prevalence of overweight and obesity among this specific group is more than 30% [3,13]. Indonesia, with a population of almost 280 million people, is the world’s fourth-largest country, and it has a fast-increasing prevalence of obesity in both children and adults in its population. According to the 2018 National Basic Health Research Survey (RISKESDAS), one out of every seven Indonesian teenagers is overweight or obese [14].

Universitas Indonesia (UI), Universitas Gajah Mada (UGM), and Universitas Airlangga (UA) are the three largest universities in Indonesia. All are members of the ASEAN University Network, which established the health promotion network to encourage a health-promoting university among its member universities. Therefore, the desire to plan intervention activities to reduce the NCD risk among the university community, especially students, is one of the main focuses. An initial step in preparation for implementing intervention activities is to identify the extent of the problem of overweight/obesity among university students, particularly those pursuing the health sciences, as they are expected to step into the role of a health peer educator. Moreover, associated factors should be identified when planning for interventions for modifiable factors associated with overweight/obesity among adolescent and young adult students.

The aim of this study was to identify the prevalence of excess body weight and associated factors among first-year health science university students as part of an endeavor to reinforce their role as agents of change especially among their peers within the health-promoting university framework. First-year students are specifically chosen as they are in the crucial period of transition from adolescent to young adult, which is a sensitive developmental period that is high risk for weight gain. Research for the evaluation of weight changes among first-year university students has garnered considerable attention [15].

## Methods

### Population and sampling

This cross-sectional study used primary data derived from first-year health sciences students of the three biggest universities in Indonesia, namely, UI in West Java province, UGM in Yogyakarta province, and UA in East Java province. The health sciences discipline comprises medicine, dentistry, psychology, pharmacy, nutrition, nursing, and public health. All first-year health sciences students who enrolled in the three universities in 2022 received an email inviting them to a health examination at the university clinic or academic hospital. For several years, all new students at UI have been required to undergo a health checkup; however, at UGM and UA, mandatory student health examinations are being implemented as part of their commitment as a Health Promoting University. Because the projected number of students in this group exceeds 2000, we determined that a power of 90% for a confidence level of 0.05 could be obtained to acquire the result and identify its associated factors. This study is part of the “Strengthening Adolescent and Young Adult-friendly Primary Care Service within University Clinics in Indonesia” (STRAYA INDO) Project, which is a collaboration project of UI, UGM, and UA with Melbourne University, Australia, and aims to strengthen health care services of university students within the ASEAN University Network (AUN) healthy university framework [16]. The study has obtained ethics approval from two universities: (i) Health Research Ethics Committee of the Faculty of Medicine Universitas Indonesia no. KET-475 UN2.F1/ETIK/PPM.00.02/2022 and (ii) Ethics Committee of Faculty of Medicine, Public Health and Nursing Universitas Gadjah Mada, number KE/1014/08/2022. Written informed consent was obtained from all participants for the use of their health examination information for this study by filling in an online form.

### Data collection and measurement

Students’ health examinations and an online survey were conducted between July 2022 and September 2022. The study outcome was excess body weight, which is a compound prevalence of overweight and obesity. The questionnaire implored demographic questions, such as sex (male/female), age (date of birth), and study major (medicine, dentistry, and others), as well as other determinant factors, including family history, eating, and exercise habit. Family history on obesity, diabetes mellitus (DM), hypertension, stroke, and heart disease were solicited pertaining to their parents as ever diagnosed by a health profession (yes/no). Questions related to eating habits asked whether the participants have regular breakfast (yes/no), the frequency of main daily meals, which was then grouped into <3 times or 3 times per day, and the frequency of fast-food consumption, which was then grouped into ≥3 times or <3 times per week. Some examples of fast food are also provided, such as fried chicken, sausages, pizza, and hamburgers, along with several types of local food. The frequency of routine exercise, which was defined as having exercise of a minimum of 30 minutes at least three times per week, was determined by asking the participant to choose never, sometimes if they did it consistently, or always if they did it consistently in most weeks for the last three months. We chose this lower-than-recommended dose of physical activity of 150 minutes per week, as it is considered to be the minimum amount of physical activity for reduced mortality and extended life expectancy, according to a large cohort study by Wen, et al [17]. Age was classified into two groups based on WHO classification (adolescents, ≤ 19 years old; young adults, > 19 years old) [18]. Fasting blood glucose (FBG) was measured at UGM and UA and analyzed at their respective academic hospital laboratories using calibrated Sysmex® XN1000. Random blood glucose (RBG) was measured at UI using the calibrated glucometer Easy Max® as part of the regular health examination for all the new UI students. A control solution test was conducted whenever a new vial of test strips was opened to ensure the meter and test strips are working properly. Increased blood glucose was determined if the students had FBG ≥ 100 mg/dL or RBG ≥ 140 mg/dL based on the criteria suggested by the American Diabetes Association [19]. Body weight was measured using a weighing scale (SECA®, Hamburg, Germany), and body height was measured using a stature meter Microtoise (GEA medical®), which was calibrated daily. Anthropometric measurements were performed by medical staff of each university following a standard protocol utilized by the Ministry of Health during their national health survey. A refresher workshop was conducted prior to data collection. Body mass index (BMI) was calculated as body weight in kilograms divided by body height in meters squared. Nutritional status was then classified based on the BMI criteria for an Asian population as follows: underweight (BMI < 18.5 kg/m^2^), normal weight (18.5–22.9 kg/m^2^), overweight (23.0–24.9 kg/m^2^), obese I (25.0–29.9 kg/m^2^), and obese II (≥30.0 kg/m^2^) [20]. An online questionnaire to capture sociodemographic data, family history, and daily habits was given to all students during data collection. The questionnaire has been used regularly at UI during the mandatory health assessment for new students. Before being adopted at UGM and UA, the questionnaire was amended through discussion among the researchers and clinic coordinators of the three universities to improve clarity and ensure similar understanding. Students completed the questionnaire on their own while waiting for medical and laboratory testing so that the data could be instantly recorded directly into a database.

### Data analysis

Data frequency was described in percentages, and associated factors were measured by the odds ratio using logistic regression. All other variables included in the multivariate logistic regression were adjusted (variables that had a p-value of <0.2 in the univariate analysis). Missing data was excluded from the analysis. A collinearity diagnostic was performed to examine multicollinearity among the independent variables included in the multivariate analysis. IBM SPSS Statistics for Windows version 20.0 (IBM Corp., Armonk, NY, USA) was used for the analysis with a p-value of 0.05 as the cutoff to determine significant risk factors associated with excess body weight (i.e., BMI ≥ 23.0 kg/m^2^).

## Results

**Fig 1:**
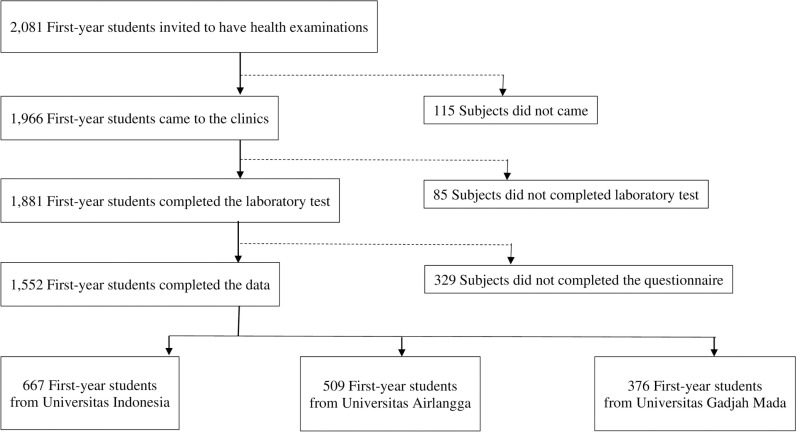
The Flow of Selection Among First-Year Students as Study Participants.

All of the 2081 first-year health sciences students from UI, UGM, and UA were initially invited for health examinations in the university clinics. Among those who were invited, 1966 came to the clinics. Among those who came for the health examination, 1881 completed the laboratory test. In total, 529 students did not complete the questionnaire; therefore, 1552 (74.6%) complete student data were used for further analysis (Fig 1). Among students with complete data, 668 (43%) were from UI, 509 (32.8%) were from UA, and 375 (24.2%) were from UGM. The characteristics of participants with incomplete data were similar to those with complete data in terms of age and sex. The highest proportions of the students were majoring in medicine (45%), and the rest were majoring in dentistry, nursing, public health, psychology, and pharmacy.

Table 1 shows the characteristics and health history of the students. The median (minimum–maximum) age of the students is 18 (16–21) years old, and the higher proportion was female (77.6%). The total prevalence of excess body weight (overweight/obese) among the students was 34.7%. The proportion of students who reported having a high-risk family history was 10.1% for stroke, 11.7% for obesity, 13.6% for heart disease, and 19.4% for diabetes. More than a quarter of the students had a family history of hypertension (26.2%). Only 27 (1.7%) of the students exhibited elevated blood glucose levels, defined as FBG > 100 mg/dL or RBG > 140 mg/dL. For questions related to their lifestyle, more than two thirds of the students stated that they had breakfast every day (79.3%), ate three times a day (65.9%), and consumed fast food less than three times a week (78.9%). Meanwhile, > 80% of students admitted to never or only occasionally engaging in routine exercise.

**Table 1:** Characteristic of the first-year health sciences students of Universitas Indonesia, Universitas Gadjah Mada, and Universitas Airlangga in 2022 (n = 1552).

Variables	*n*	%
**Age**		
**Adolescents (≤19 years)**	1519	**97.9**
**Young adults (>19 years)**	33	2.1
**Sex**		
**Male**	347	22.4
**Female**	1205	**77.6**
**Study major**		
**Medicine/dentistry**	789	**50.9**
**Other**	763	49.1
**Nutritional status**		
**Underweight (BMI < 18.5 kg/m**^**2**^)	340	21.9
**Normo-weight (BMI 18.5–22.9 kg/m**^**2**^)	674	43.4
**Overweight (BMI 23.0–24.9 kg/m**^**2**^)	205	13.2
**Obese I (BMI 25.0–29.9 kg/m**^**2**^)	235	15.1
**Obese II (BMI ≥ 30.0 kg/m**^**2**^)	98	6.3
**Family history of obesity**		
**Yes**	181	**11.7**
**No**	1371	88.3
**Family history of diabetes**		
**Yes**	301	**19.4**
**No**	1251	80.6
**Family history of hypertension**		
**Yes**	407	**26.2**
**No**	1145	73.8
**Family history of stroke**		
**Yes**	156	**10.1**
**No**	1396	89.9
**Family history of heart disease**		
**Yes**	211	**13.6**
**No**	1341	86.4
**Increased blood glucose**		
**Yes (FBG > 100 mg/dL or RBG > 140 mg/dL)**	27	1.7
**No**	1525	**98.3**
**Daily breakfast**		
**No**	322	20.7
**Yes**	1230	**79.3**
**Daily meal**		
**<3 times/day**	529	34.1
**3 times/day**	1023	**65.9**
**Fast food consumption**		
**≥3 times/week**	327	21.1
**<3 times/week**	1225	**78.9**
**Routine exercise**		
**Never-sometimes**	1261	**81.3**
**Always**	291	18.8

BMI, body mass index; FBG, fasting blood glucose; RBG, random blood glucose.

Table 2 shows factors associated with excess body weight based on the bivariate analysis. Excess body weight was significantly associated with being male, majoring in medicine or dentistry, having a family history of obesity or diabetes, not eating breakfast daily, and eating <3 meals/day.

**Table 2:** Association (Unadjusted) Between Excess Body Weight and Their Determinant Factors Among First-Year Health Sciences Students of Universitas Indonesia, Universitas Gadjah Mada, and Universitas Airlangga in 2022 (n = 1552).

Variables	Excess body weight				Crude OR	*p* value	95% Confidence Interval
	Yes		No				
	N	%	N	%			
Age							
**Adolescents (≤19 years)**	532	35.0	987	65.0	1.10	0.050	0.17–1.01
**Young adults (>19 years)**	6	18.2	27	81.8		Reference	
**Sex**							
**Male**	180	51.9	167	48.1	2.55	**<0.001**	2.00–3.25
**Female**	358	29.7	847	70.3		Reference	
**Study major**							
**Medicine/dentistry**	297	37.6	492	62.4	1.31	**0.012**	1.06–1.61
**Other**	241	31.6	522	68.4		Reference	
**Family history of obesity**							
**Yes**	108	59.7	73	40.3	3.24	**<0.001**	2.35–4.45
**No**	430	31.4	941	68.6		Reference	
**Family history of diabetes**							
**Yes**	121	40.2	180	59.8	1.34	**0.025**	1.04–1.74
**No**	417	33.3	834	66.7		Reference	
**Family history of hypertension**							
**Yes**	156	38.3	251	61.7	1.24	0.071	0.98–1.57
**No**	382	33.4	763	66.6		Reference	
**Family history of stroke**							
**Yes**	61	39.1	95	60.9	1.24	0.220	0.88–1.74
**No**	477	34.2	919	65.8		Reference	
**Family history of heart disease**							
**Yes**	76	36.0	135	64.0	1.07	0.657	0.79–1.45
**No**	462	34.5	879	65.5		Reference	
**Increased blood glucose**							
**Yes (FBG > 100 mg/dL or RBG > 140 mg/dL)**	9	33.3	18	66.7	0.94	0.883	0.42–2.11
**No**	529	34.7	996	65.3		Reference	
**Daily breakfast**							
**No**	137	42.5	185	57.5	1.53	**0.001**	1.19–1.97
**Yes**	401	32.6	829	67.4		Reference	
**Daily meal**							
**<3 times/day**	210	39.7	319	60.3	1.39	**0.003**	1.12–1.73
**3 times/day**	328	32.1	695	67.9		Reference	
**Fast food**							
**≥3 times/week**	109	33.3	218	66.7	0.93	0.569	0.72–1.20
**<3 times/week**	429	35.0	796	65.0		Reference	
**Routine exercise**							
**Never sometimes**	423	33.5	838	66.5	0.77	0.054	0.59–1.00
**Always**	115	39.5	176	60.5		Reference	

Table 3 shows factors independently associated with overweight/obesity based on the multivariate logistic regression analysis. From the multivariate analysis, the independent factors related to overweight or obesity in students were the male sex (adjusted odds ratio [AOR], 2.58; 95% CI 1.99–3.35), having a family history of obesity (AOR, 2.91; 95% CI 2.09–4.06), and eating <3 meals/day (AOR, 1.40; 95% CI 1.10–1.80). We found no indication of multicollinearity among independent variables.

**Table 3:** Factors Independently Associated with Overweight/Obesity Among the First-Year Health Sciences Students of Universitas Indonesia, Universitas Gadjah Mada, and Universitas Airlangga in 2022 (n = 1552).

Variables	Beta	SE	Adjusted OR	P-value	95% confidence interval
**Male**	0.95	0.13	2.58	<0.001	1.99–3.35
**Have a family history of obesity**	1.07	0.17	2.91	<0.001	2.09–4.06
**Daily meal < 3 × /day**	0.34	0.12	1.40	<0.001	1.10–1.80

## Discussion

This study showed that 34.7% of the respondents were in excess body weight (overweight/obese). Being male, having a family history of obesity, and eating less than three meals per day increased the risk of becoming overweight/obese. Respondents were new students in 2022 who were experiencing online learning for the last two years during the COVID-19 pandemic. The prevalence of overweight/obesity in our respondents aged 16–21 years was high compared with the national overweight/obesity rate of 13.5% for ages 16–18 years and 26.6% for ages >18 years [14]. Lifestyle during the pandemic, such as the increased in sedentary behaviors because of participation in online learning, might have a role in increasing the prevalence of overweight or obesity among our respondents who have just graduated from high school and are newly entering the university. Despite the current absence of data related to the incidence of obesity in Indonesia during the pandemic, studies in North Africa [21], India [22], the Netherlands [23], and Germany [24] have reported a considerable increase in BMI among adolescents during the pandemic. Furthermore, the three institutions that participated in this study were located in Indonesia’s three largest cities, which also have the greatest prevalence of overweight/obesity and NCDs in the country. A study among first-year medical students of UI in 2018 reported that the overweight/obesity prevalence was 48.7%, which was also high [25].

Male students are a minority in the health sciences major (22.4%); however, their risk of overweight/obesity is higher as they are 2.6 times more likely to be at risk for overweight/obesity than female students. Studies of the overweight/obesity prevalence among adolescents and young adults in various communities have shown differences in the prevalence of overweight/obesity between men and women. Some studies have reported a higher prevalence in women [26–28], others have shown a higher prevalence of overweight/obesity in men [29], and other studies have reported no significant difference in the prevalence of overweight/obesity between men and women [30]. This phenomenon is also supported by a systematic review by Munusamy and Shanmugam in 2022 [31]. However, studies on health sciences university students in Saudi Arabia [32], Peru [13], and Turkey [33] reported that male students are more likely to be overweight/obese. We expect a complex multivariate relationship between sex and overweight/obesity. However, women were reported to exhibit greater health-related behavior [34] and body image awareness than men [35].

In our study, students with a family history of obesity have almost three times the risk of having excess body weight compared with those without a family history of obesity. This was also observed in the previous studies in Saudi Arabia [32], Peru [13] and Turkey [33]. Having a family history of obesity and hypertension, type 2 diabetes mellitus, and coronary heart disease is an important risk factor for a precocious onset of obesity in childhood and influences the severity of obesity [36]. In our study, the association between family history of diabetes and hypertension to excess body weight is also observed in the bivariate analysis. However, in multivariate analysis, family history of obesity is the only family factor that remains the independent factor, as the existence of all family factors might be related to each other.

We found that those who are eating less than three meals a day have a 1.4 times higher risk of becoming overweight/obese than those who are eating three meals a day. A meta-analysis by Kaisari et al., which involved 18,849 children and adolescents (aged 2–19 years), concluded that a higher eating frequency was associated with a lower risk of overweight/obesity in children and adolescents, especially in boys [37]. A meta-analysis by Wang et al., which was published in 2016 and involved 65,742 adults (age, > 20 years), also revealed that increased eating frequency was associated with 0.83-time lower odds of developing obesity, although associated with higher energy intake [38]. However, in terms of the participant age (age 16–21 years), the present study was similar to that of Kaisari et al. Both meta-analyses involved only observational studies with substantial heterogeneity, including the definition of eating frequency. Some studies included snacks in the counting, whereas others did not. However, the beneficial effect of increased eating frequency on insulin metabolism is suggested as a potential biological mechanism, as increased eating frequency attenuates a series of postprandial metabolic and endocrine responses to dietary intake [39–40]. Moreover, the nutrition component of a diet needs to be assessed further by evaluating dietary patterns based on the national dietary guidelines rather than just recommending an improvement in the eating frequency.

In the bivariate analysis, those who have a habit of skipping breakfast have a 1.5 times higher risk of becoming overweight/obese compared with those who regularly eat breakfast, although this relationship faded in the multivariate analysis. Two meta-analyses have already been conducted on this topic, and both concluded that skipping breakfast increases the risk of overweight/obesity among different age groups, sexes, regions, and economic conditions [41–42].

When university graduates enter the community, they are expected to become the agents of change for healthy life, particularly in the field of health sciences. The expectation for health sciences university students still in their adolescence and young adult group is high because if they are unhealthy, they will leave the wrong impression on the general population.

This study has some limitations. First, the study only included health sciences students from the three most prominent universities located on the most populated island, where the socio-economic conditions might not reflect the condition of the health science students in other settings. Second, the timing of data collection, which was conducted two years after the COVID-19 pandemic started, might influence the high prevalence of overweight and obesity observed in this study. Furthermore, some of the variable measures, such as those for eating habits and exercise, were not standard, as the questionnaire needed to be practical to be implemented for many students. During the health examination period, the universities are still limiting offline activities. Hence, we need to keep the examination as short as possible.

## Conclusion

The prevalence of overweight/obesity is quite high among first-year health sciences university students. Besides identifying some nonmodifiable risk factors of overweight/obesity, this study also identified some modifiable factors such as eating frequency and breakfast habits, which could be promoted among the students. The health sciences faculties should design an education curriculum which not only ensures that the students can provide health care to the communities in the future but also apply the knowledge to improve their health.

## Supporting information

S1 Table Characteristic distribution of the first-year health science students of three universities in 2022. (DOCX)

S2 Table Central tendency measures of age, body weight, height, Body Mass Index, and blood glucose of the first-year health science students of three Indonesian universities in 2022 (n = 2081). (DOCX)
