# Supplementary material for: Excess body weight and its associated factors among first-year health sciences university students in Indonesia
Source: PLoS One. 2025 May 29;20(5):e0322773. doi: 10.1371/journal.pone.0322773 (PMC12122023; doi:10.1371/journal.pone.0322773)
Supplement: S1 Table — (DOCX) [file pone.0322773.s001.docx]

**S1 Table. Characteristic distribution of the first-year health science students of three universities in 2022**

| **Variables** | **Uni1** | | **Uni2** | | **Uni3** | |
| --- | --- | --- | --- | --- | --- | --- |
|  | **n** | **%** | **n** | **%** | **n** | **%** |
| **Age** |  |  |  |  |  |  |
| **Adolescents (≤19 years)** | 978 | **98.1** | 322 | **80.5** | 671 | **98.1** |
| **Young adults (>19 years)** | 19 | 1.9 | 78 | 19.5 | 13 | 1.9 |
| **Sex** |  |  |  |  |  |  |
| **Male** | 217 | 21.8 | 86 | 21.5 | 184 | 26.9 |
| **Female** | 780 | **78.2** | 314 | **78.5** | 500 | **73.1** |
| **Study major (Category)** |  |  |  |  |  |  |
| **Medicine/dentistry** | 392 | **39.3** | 213 | **53.3** | 413 | **60.4** |
| **Other** | 605 | 60.7 | 187 | 46.8 | 271 | 39.6 |
| **Nutritional status** |  |  |  |  |  |  |
| **Underweight (BMI < 18.5 kg/m^2^)** | 187 | 18.8 | 99 | 24.8 | 136 | 19.9 |
| **Normo-weight (BMI 18.5–22.9 kg/m^2^)** | 417 | **41.8** | 183 | **45.8** | 237 | **34.6** |
| **Overweight (BMI 23.0–24.9 kg/m^2^)** | 118 | 11.8 | 50 | 12.5 | 89 | 13.0 |
| **Obese I (BMI 25.0–29.9 kg/m^2^)** | 136 | 13.6 | 42 | 10.5 | 108 | 15.8 |
| **Obese II (BMI >30.0 kg/m^2^)** | 56 | 5.6 | 17 | 4.3 | 43 | 6.5 |
| **Missing** | 83 | 8.3 | 9 | 2.3 | 69 | 10.1 |
| **Family history of obesity** |  |  |  |  |  |  |
| **Yes** | 98 | **9.8** | 35 | **8.8** | 98 | **14.3** |
| **No** | 897 | 90.0 | 365 | 91.3 | 517 | 75.6 |
| **Missing** | 2 | 0.2 | - | - | 69 | 10.1 |
| **Family history of diabetes** |  |  |  |  |  |  |
| **Yes** | 139 | **13.9** | 83 | **20.8** | 142 | **20.8** |
| **No** | 856 | 85.9 | 317 | 79.3 | 473 | 69.2 |
| **Missing** | 2 | 0.2 | - | - | 69 | 10.1 |
| **Family history of hypertension** |  |  |  |  |  |  |
| **Yes** | 279 | **28.0** | 112 | **28.0** | 155 | **22.7** |
| **No** | 716 | 71.8 | 288 | 72.0 | 460 | 67.3 |
| **Missing** | 2 | 0.2 | - | - | 69 | 10.1 |
| **Family history of stroke** |  |  |  |  |  |  |
| **Yes** | 89 | **8.9** | 36 | **9.0** | 80 | **11.7** |
| **No** | 906 | 90.9 | 364 | 91.0 | 535 | 78.2 |
| **Missing** | 2 | 0.2 | - | - | 69 | 10.1 |
| **Family history of heart disease** |  |  |  |  |  |  |
| **Yes** | 129 | **12.9** | 47 | **11.8** | 93 | **13.6** |
| **No** | 866 | 86.9 | 353 | 88.3 | 522 | 76.3 |
| **Missing** | 2 | 0.2 | - | - | 69 | 10.1 |
| **Increased blood glucose** |  |  |  |  |  |  |
| **Yes (FBG > 100 mg/dL or RBG > 140 mg/dL)** | 23 | 2.3 | 5 | 1.3 | 3 | 0.4 |
| **No** | 886 | **98.9** | 386 | **96.5** | 578 | **84.9** |
| **Missing** | 88 | 8.8 | 9 | 2.3 | 103 | 15.1 |
| **Daily breakfast** |  |  |  |  |  |  |
| **No** | 159 | 15.9 | 68 | 17.0 | 177 | 25.9 |
| **Yes** | 835 | **83.8** | 322 | **83.0** | 428 | **62.6** |
| **Missing** | 3 | 0.3 | - | - | 79 | 11.5 |
| **Daily meal (Category)** |  |  |  |  |  |  |
| **<3 times/day** | 277 | 27.8 | 121 | 30.3 | 269 | 39.3 |
| **3 times/day** | 692 | **69.4** | 271 | **67.8** | 326 | **47.7** |
| **Missing** | 28 | 2.8 | 8 | 2.0 | 89 | 13.0 |
| **Fast food consumption** |  |  |  |  |  |  |
| **≥3 times/week** | 175 | 17.6 | 99 | 24.8 | 144 | 21.1 |
| **<3 times/week** | 819 | **82.1** | 301 | **75.3** | 461 | **67.4** |
| **Missing** | 3 | 0.3 | - | - | 79 | 11.5 |
| **Routine exercise** |  |  |  |  |  |  |
| **Never** | 99 | 9.9 | 14 | 3.5 | 78 | 11.4 |
| **Sometimes** | 721 | **72.3** | 298 | **74.5** | 408 | **59.6** |
| **Always** | 174 | 17.5 | 88 | 22.0 | 119 | 17.4 |
| **Missing** | 3 | 0.3 | - | - | 79 | 11.5 |
