# Supplementary material for: Excess body weight and its associated factors among first-year health sciences university students in Indonesia
Source: PLoS One. 2025 May 29;20(5):e0322773. doi: 10.1371/journal.pone.0322773 (PMC12122023; doi:10.1371/journal.pone.0322773)
Supplement: S2 Table — (DOCX) [file pone.0322773.s002.docx]

**S2 Table. Central tendency measures of age, body weight, height, Body Mass Index, and blood glucose of the first-year health science students of three Indonesian universities in 2022 (n = 2081).**

| **Variables** | **Uni1** | | | | **Uni2** | | | **Uni3** | | |
| --- | --- | --- | --- | --- | --- | --- | --- | --- | --- | --- |
|  | **n** | **Mean (SD)** | **Median (Min-Max)** | **n** | | **Mean (SD)** | **Median (Min-Max)** | **n** | **Mean (SD)** | **Median (Min-Max)** |
| **Age** | 997 | 18.02 (0.66) | 18 (16 – 21) | 400 | | 18.07 (0.66) | 18 (16 – 20) | 684 | 17.95 (0.65) | 18 (16 – 20) |
| **Weight** | 936 | 57.52 (14.03) | 54.50 (30 – 117) | 400 | | 55.84 (13.01) | 53 (35 – 115) | 649 | 58.36 (15.27) | 55 (30 – 130) |
| **Height** | 933 | 161.05 (7.70) | 160 (140 – 193) | 400 | | 160.67 (7.38) | 160 (140 – 184) | 634 | 160.77 (7.96) | 160 (127 – 185) |
| **Body Mass Index (BMI)** | 933 | 22.04 (4.43) | 21.22 (12.98 - 42.76) | 400 | | 21.51 (4.18) | 20.82 (14.68 - 44.92) | 633 | 22.43 (4.77) | 21.71 (13.67 - 44.58) |
| **Fasting Blood Glucose (FBG)** | - | - | - | 391 | | 89.70 (8.29) | 90 (67 – 129) | 581 | 78.74 (10.97) | 78 (52 – 249) |
| **Random Blood Glucose (RBG)** | 909 | 102.10 (17.63) | 98 (50 – 277) |  | | - | - |  | - | - |
